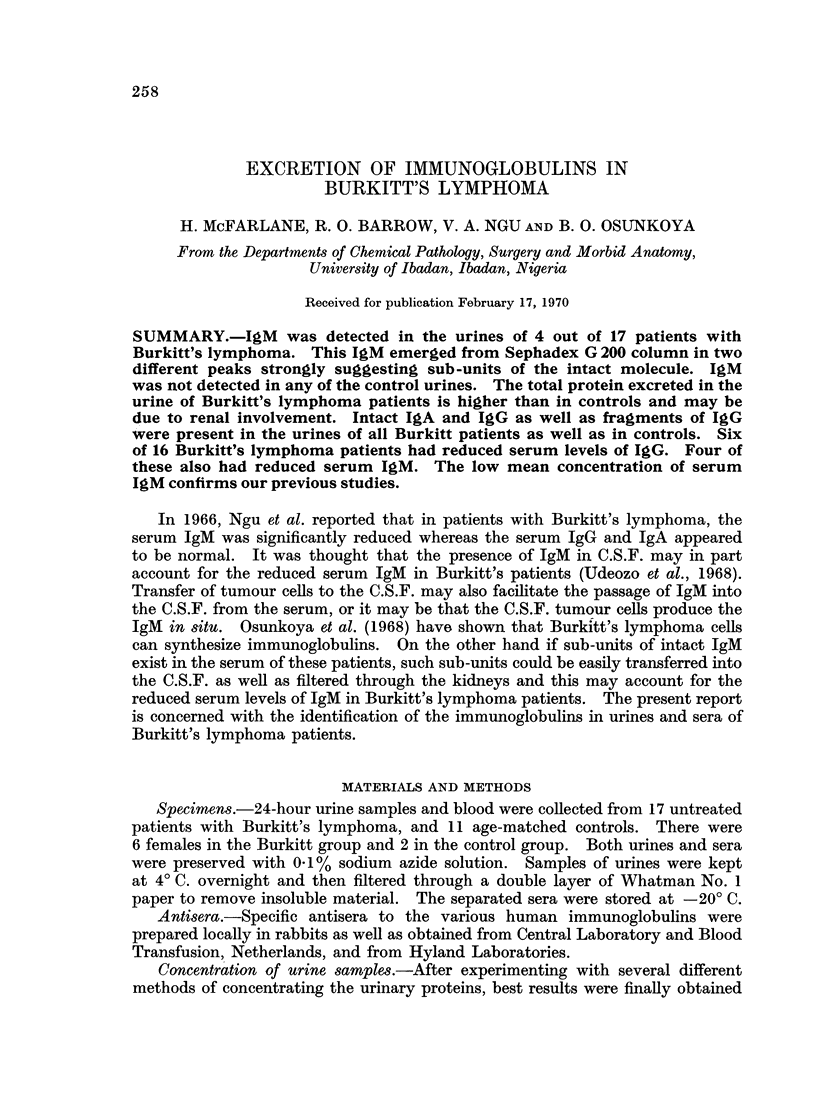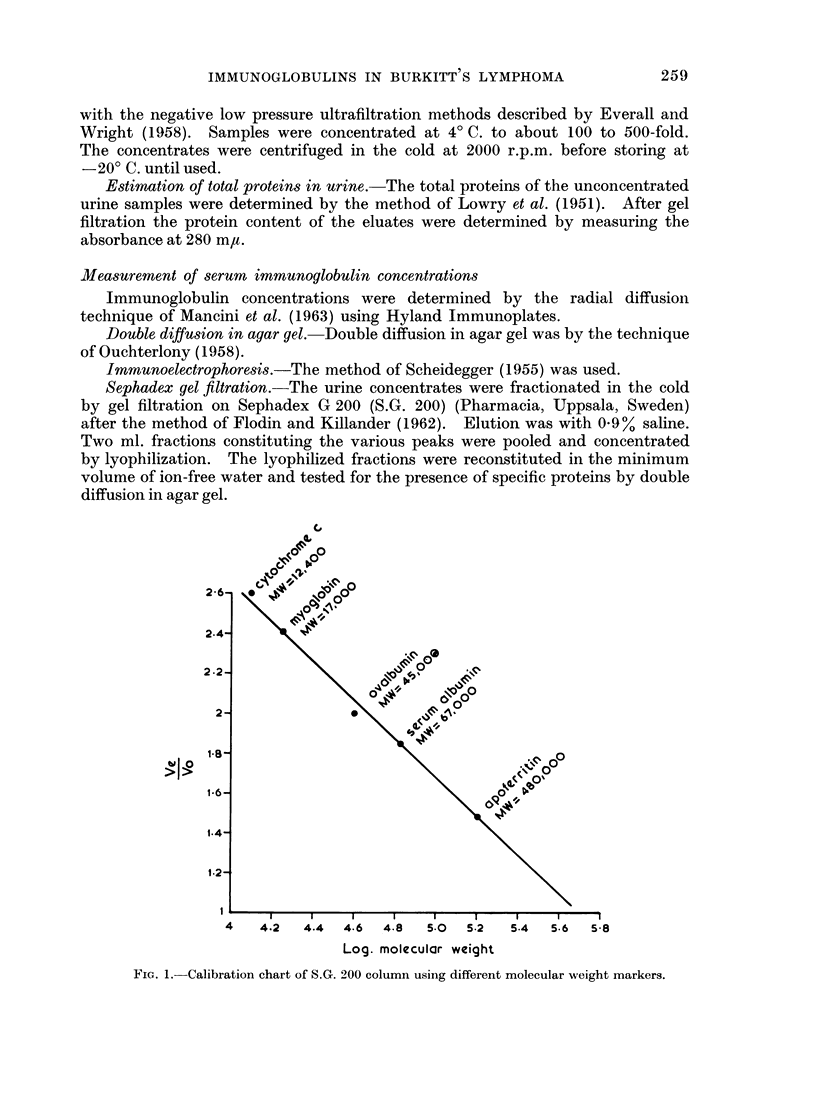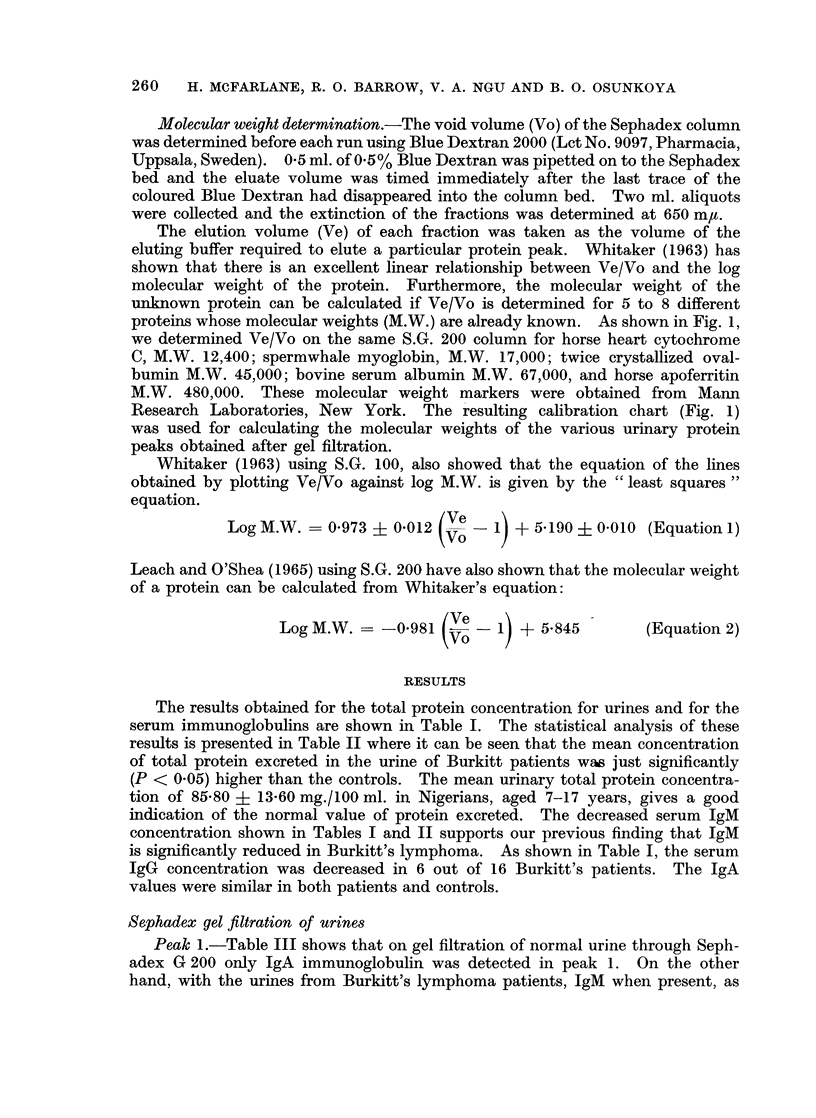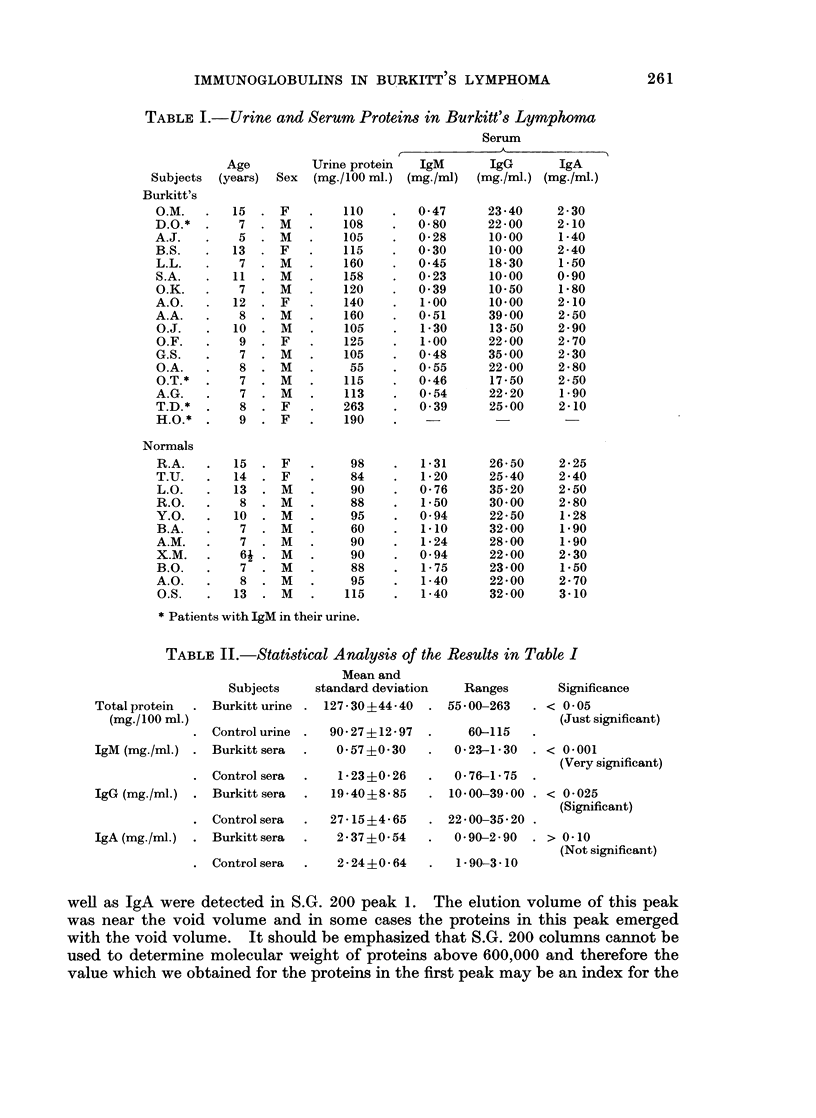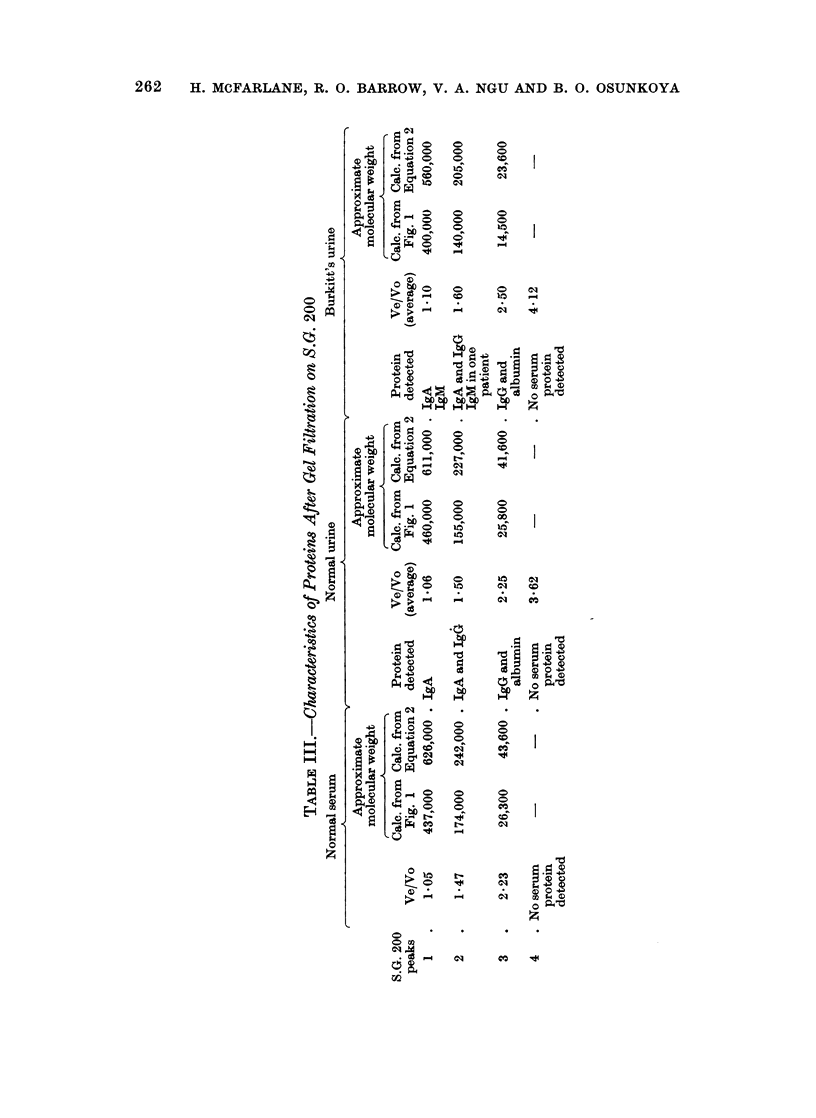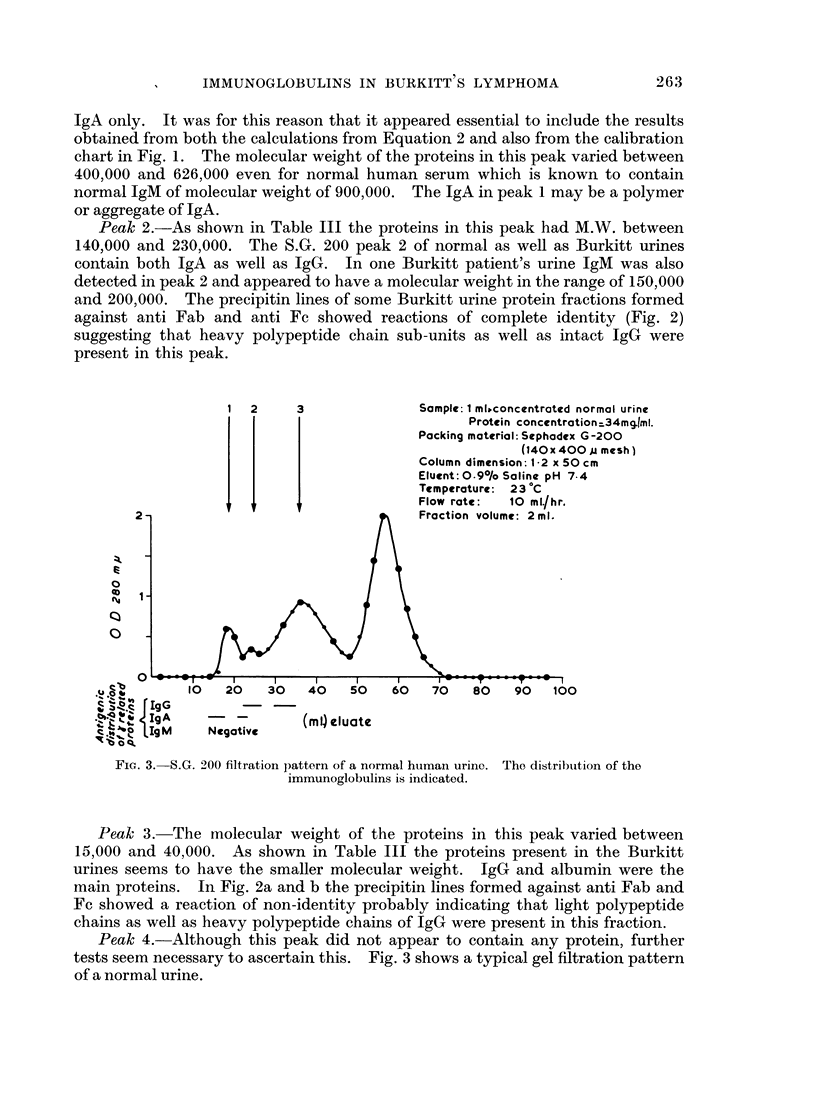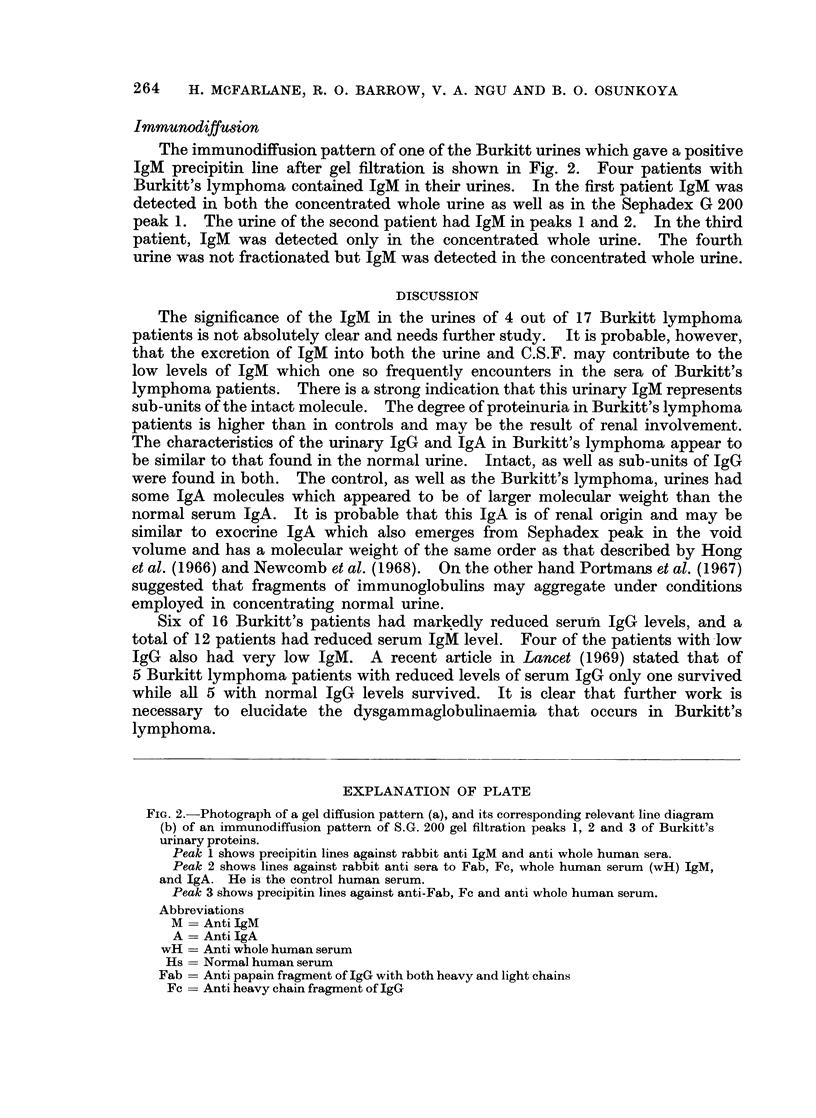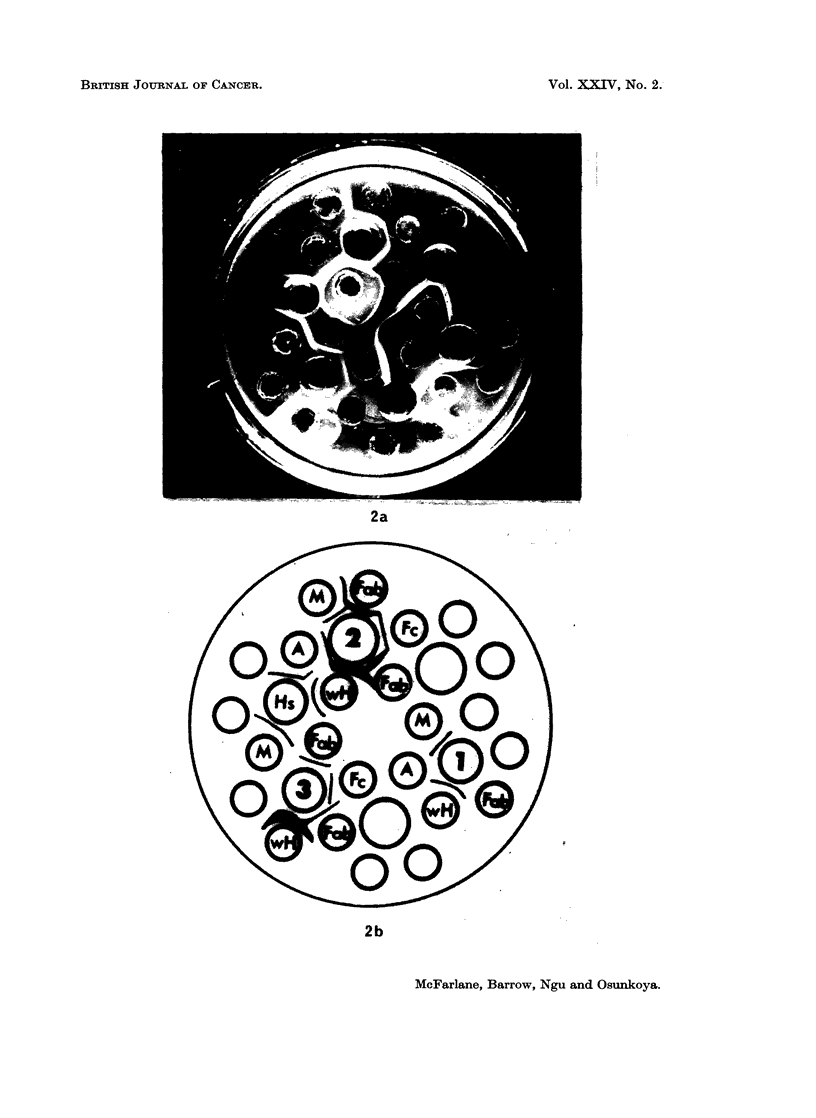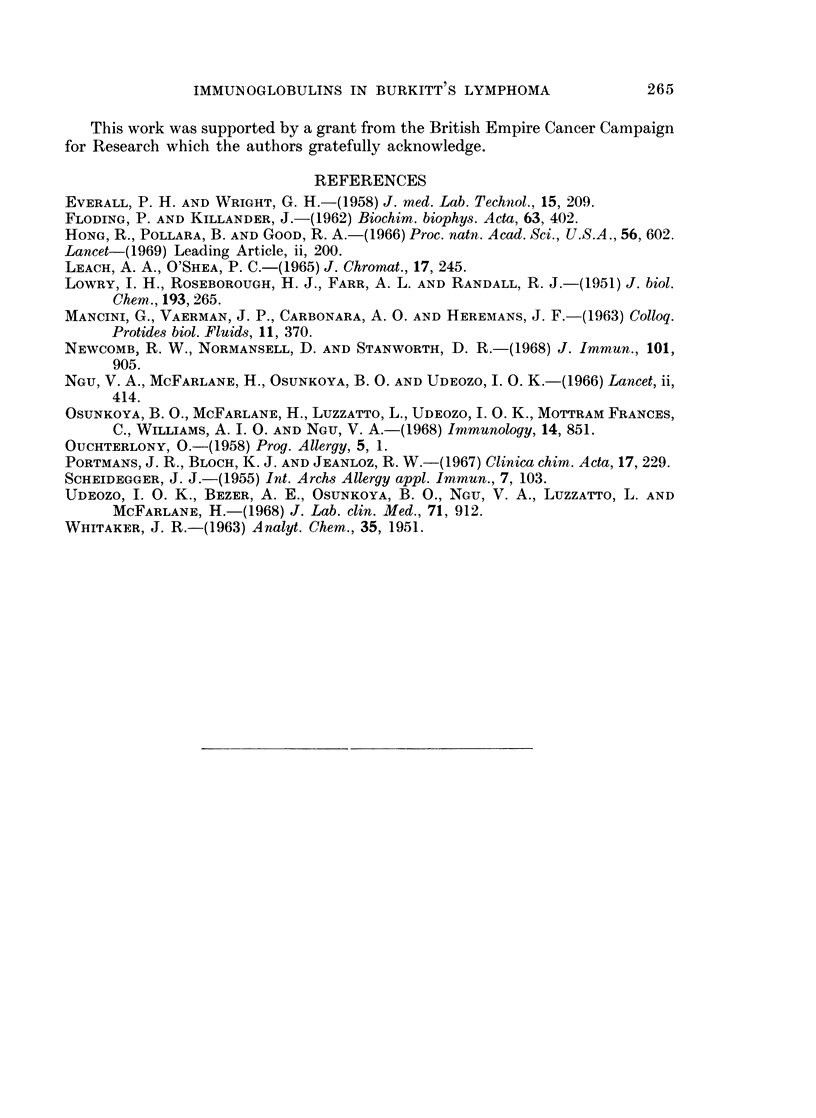# Excretion of Immunoglobulins in Burkitt's Lymphoma

**DOI:** 10.1038/bjc.1970.29

**Published:** 1970-06

**Authors:** H. McFarlane, R. O. Barrow, V. A. Ngu, B. O. Osunkoya

## Abstract

**Images:**


### EXCRETION OF IMMUNOGLOBULINS IN BURKITT'S LYMPHOMA

H. McFARLANE, R. O. BARROW, V. A. NGU AND B. O. OSUNKOYA

*From the Departments of Chemical Pathology, Surgery and Morbid Anatomy, University of Ibadan, Ibadan, Nigeria*

Received for publication February 17, 1970

**SUMMARY.—IgM was detected in the urines of 4 out of 17 patients with Burkitt's lymphoma. This IgM emerged from Sephadex G 200 column in two different peaks strongly suggesting sub-units of the intact molecule. IgM was not detected in any of the control urines. The total protein excreted in the urine of Burkitt's lymphoma patients is higher than in controls and may be due to renal involvement. Intact IgA and IgG as well as fragments of IgG were present in the urines of all Burkitt patients as well as in controls. Six of 16 Burkitt's lymphoma patients had reduced serum levels of IgG. Four of these also had reduced serum IgM. The low mean concentration of serum IgM confirms our previous studies.**

In 1966, Ngu *et al.* reported that in patients with Burkitt's lymphoma, the serum IgM was significantly reduced whereas the serum IgG and IgA appeared to be normal. It was thought that the presence of IgM in C.S.F. may in part account for the reduced serum IgM in Burkitt's patients (Udeozo *et al.*, 1968). Transfer of tumour cells to the C.S.F. may also facilitate the passage of IgM into the C.S.F. from the serum, or it may be that the C.S.F. tumour cells produce the IgM *in situ*. Osunkoya *et al.* (1968) have shown that Burkitt's lymphoma cells can synthesize immunoglobulins. On the other hand if sub-units of intact IgM exist in the serum of these patients, such sub-units could be easily transferred into the C.S.F. as well as filtered through the kidneys and this may account for the reduced serum levels of IgM in Burkitt's lymphoma patients. The present report is concerned with the identification of the immunoglobulins in urines and sera of Burkitt's lymphoma patients.

### MATERIALS AND METHODS

*Specimens.*—24-hour urine samples and blood were collected from 17 untreated patients with Burkitt's lymphoma, and 11 age-matched controls. There were 6 females in the Burkitt group and 2 in the control group. Both urines and sera were preserved with 0·1% sodium azide solution. Samples of urines were kept at 4° C. overnight and then filtered through a double layer of Whatman No. 1 paper to remove insoluble material. The separated sera were stored at —20° C.

*Antisera.*—Specific antisera to the various human immunoglobulins were prepared locally in rabbits as well as obtained from Central Laboratory and Blood Transfusion, Netherlands, and from Hyland Laboratories.

*Concentration of urine samples.*—After experimenting with several different methods of concentrating the urinary proteins, best results were finally obtained with the negative low pressure ultrafiltration methods described by Everall and Wright (1958). Samples were concentrated at 4° C. to about 100 to 500-fold. The concentrates were centrifuged in the cold at 2000 r.p.m. before storing at —20° C. until used.

*Estimation of total proteins in urine.*—The total proteins of the unconcentrated urine samples were determined by the method of Lowry *et al.* (1951). After gel filtration the protein content of the eluates were determined by measuring the absorbance at 280 m$\mu$.

*Measurement of serum immunoglobulin concentrations*

Immunoglobulin concentrations were determined by the radial diffusion technique of Mancini *et al.* (1963) using Hyland Immunoplates.

*Double diffusion in agar gel.*—Double diffusion in agar gel was by the technique of Ouchterlony (1958).

*Immunoelectrophoresis.*—The method of Scheidegger (1955) was used.

*Sephadex gel filtration.*—The urine concentrates were fractionated in the cold by gel filtration on Sephadex G 200 (S.G. 200) (Pharmacia, Uppsala, Sweden) after the method of Flodin and Killander (1962). Elution was with 0·9% saline. Two ml. fractions constituting the various peaks were pooled and concentrated by lyophilization. The lyophilized fractions were reconstituted in the minimum volume of ion-free water and tested for the presence of specific proteins by double diffusion in agar gel.

FIG. 1.—Calibration chart of S.G. 200 column using different molecular weight markers.

*Molecular weight determination.*—The void volume (Vo) of the Sephadex column was determined before each run using Blue Dextran 2000 (Lct No. 9097, Pharmacia, Uppsala, Sweden). 0·5 ml. of 0·5% Blue Dextran was pipetted on to the Sephadex bed and the eluate volume was timed immediately after the last trace of the coloured Blue Dextran had disappeared into the column bed. Two ml. aliquots were collected and the extinction of the fractions was determined at 650 m$\mu$.

The elution volume (Ve) of each fraction was taken as the volume of the eluting buffer required to elute a particular protein peak. Whitaker (1963) has shown that there is an excellent linear relationship between Ve/Vo and the log molecular weight of the protein. Furthermore, the molecular weight of the unknown protein can be calculated if Ve/Vo is determined for 5 to 8 different proteins whose molecular weights (M.W.) are already known. As shown in Fig. 1, we determined Ve/Vo on the same S.G. 200 column for horse heart cytochrome C, M.W. 12,400; spermwhale myoglobin, M.W. 17,000; twice crystallized ovalbumin M.W. 45,000; bovine serum albumin M.W. 67,000, and horse apoferritin M.W. 480,000. These molecular weight markers were obtained from Mann Research Laboratories, New York. The resulting calibration chart (Fig. 1) was used for calculating the molecular weights of the various urinary protein peaks obtained after gel filtration.

Whitaker (1963) using S.G. 100, also showed that the equation of the lines obtained by plotting Ve/Vo against log M.W. is given by the " least squares " equation.

$$\text{Log M.W.} = 0{\cdot}973 \pm 0{\cdot}012 \left(\frac{\text{Ve}}{\text{Vo}} - 1\right) + 5{\cdot}190 \pm 0{\cdot}010 \quad \text{(Equation 1)}$$

Leach and O'Shea (1965) using S.G. 200 have also shown that the molecular weight of a protein can be calculated from Whitaker's equation:

$$\text{Log M.W.} = -0{\cdot}981 \left(\frac{\text{Ve}}{\text{Vo}} - 1\right) + 5{\cdot}845 \quad \text{(Equation 2)}$$

### RESULTS

The results obtained for the total protein concentration for urines and for the serum immunoglobulins are shown in Table I. The statistical analysis of these results is presented in Table II where it can be seen that the mean concentration of total protein excreted in the urine of Burkitt patients was just significantly ($P < 0{\cdot}05$) higher than the controls. The mean urinary total protein concentration of 85·80 ± 13·60 mg./100 ml. in Nigerians, aged 7–17 years, gives a good indication of the normal value of protein excreted. The decreased serum IgM concentration shown in Tables I and II supports our previous finding that IgM is significantly reduced in Burkitt's lymphoma. As shown in Table I, the serum IgG concentration was decreased in 6 out of 16 Burkitt's patients. The IgA values were similar in both patients and controls.

### *Sephadex gel filtration of urines*

*Peak* 1.—Table III shows that on gel filtration of normal urine through Sephadex G 200 only IgA immunoglobulin was detected in peak 1. On the other hand, with the urines from Burkitt's lymphoma patients, IgM when present, as

TABLE I.—*Urine and Serum Proteins in Burkitt's Lymphoma*

| Subjects | Age (years) | Sex | Urine protein (mg./100 ml.) | Serum IgM (mg./ml) | Serum IgG (mg./ml.) | Serum IgA (mg./ml.) |
|---|---|---|---|---|---|---|
| Burkitt's | | | | | | |
| O.M. | 15 | F | 110 | 0·47 | 23·40 | 2·30 |
| D.O.* | 7 | M | 108 | 0·80 | 22·00 | 2·10 |
| A.J. | 5 | M | 105 | 0·28 | 10·00 | 1·40 |
| B.S. | 13 | F | 115 | 0·30 | 10·00 | 2·40 |
| L.L. | 7 | M | 160 | 0·45 | 18·30 | 1·50 |
| S.A. | 11 | M | 158 | 0·23 | 10·00 | 0·90 |
| O.K. | 7 | M | 120 | 0·39 | 10·50 | 1·80 |
| A.O. | 12 | F | 140 | 1·00 | 10·00 | 2·10 |
| A.A. | 8 | M | 160 | 0·51 | 39·00 | 2·50 |
| O.J. | 10 | M | 105 | 1·30 | 13·50 | 2·90 |
| O.F. | 9 | F | 125 | 1·00 | 22·00 | 2·70 |
| G.S. | 7 | M | 105 | 0·48 | 35·00 | 2·30 |
| O.A. | 8 | M | 55 | 0·55 | 22·00 | 2·80 |
| O.T.* | 7 | M | 115 | 0·46 | 17·50 | 2·50 |
| A.G. | 7 | M | 113 | 0·54 | 22·20 | 1·90 |
| T.D.* | 8 | F | 263 | 0·39 | 25·00 | 2·10 |
| H.O.* | 9 | F | 190 | — | — | — |
| Normals | | | | | | |
| R.A. | 15 | F | 98 | 1·31 | 26·50 | 2·25 |
| T.U. | 14 | F | 84 | 1·20 | 25·40 | 2·40 |
| L.O. | 13 | M | 90 | 0·76 | 35·20 | 2·50 |
| R.O. | 8 | M | 88 | 1·50 | 30·00 | 2·80 |
| Y.O. | 10 | M | 95 | 0·94 | 22·50 | 1·28 |
| B.A. | 7 | M | 60 | 1·10 | 32·00 | 1·90 |
| A.M. | 7 | M | 90 | 1·24 | 28·00 | 1·90 |
| X.M. | 6½ | M | 90 | 0·94 | 22·00 | 2·30 |
| B.O. | 7 | M | 88 | 1·75 | 23·00 | 1·50 |
| A.O. | 8 | M | 95 | 1·40 | 22·00 | 2·70 |
| O.S. | 13 | M | 115 | 1·40 | 32·00 | 3·10 |

* Patients with IgM in their urine.

TABLE II.—*Statistical Analysis of the Results in Table I*

| | Subjects | Mean and standard deviation | Ranges | Significance |
|---|---|---|---|---|
| Total protein (mg./100 ml.) | Burkitt urine | 127·30±44·40 | 55·00–263 | < 0·05 (Just significant) |
| | Control urine | 90·27±12·97 | 60–115 | |
| IgM (mg./ml.) | Burkitt sera | 0·57±0·30 | 0·23–1·30 | < 0·001 (Very significant) |
| | Control sera | 1·23±0·26 | 0·76–1·75 | |
| IgG (mg./ml.) | Burkitt sera | 19·40±8·85 | 10·00–39·00 | < 0·025 (Significant) |
| | Control sera | 27·15±4·65 | 22·00–35·20 | |
| IgA (mg./ml.) | Burkitt sera | 2·37±0·54 | 0·90–2·90 | > 0·10 (Not significant) |
| | Control sera | 2·24±0·64 | 1·90–3·10 | |

well as IgA were detected in S.G. 200 peak 1. The elution volume of this peak was near the void volume and in some cases the proteins in this peak emerged with the void volume. It should be emphasized that S.G. 200 columns cannot be used to determine molecular weight of proteins above 600,000 and therefore the value which we obtained for the proteins in the first peak may be an index for the

TABLE III.—*Characteristics of Proteins After Gel Filtration on S.G. 200*

| | Normal serum | | | | Normal urine | | | | Burkitt's urine | | | |
|---|---|---|---|---|---|---|---|---|---|---|---|---|
| | | Approximate molecular weight | | | | Approximate molecular weight | | | | Approximate molecular weight | | |
| S.G. 200 peaks | Ve/Vo | Calc. from Fig. 1 | Calc. from Equation 2 | Protein detected | Ve/Vo (average) | Calc. from Fig. 1 | Calc. from Equation 2 | Protein detected | Ve/Vo (average) | Calc. from Fig. 1 | Calc. from Equation 2 | |
| 1 . | 1·05 | 437,000 | 626,000 . | IgA | 1·06 | 460,000 | 611,000 . | IgA IgM | 1·10 | 400,000 | 560,000 | |
| 2 . | 1·47 | 174,000 | 242,000 . | IgA and IgG | 1·50 | 155,000 | 227,000 . | IgA and IgG IgM in one patient | 1·60 | 140,000 | 205,000 | |
| 3 . | 2·23 | 26,300 | 43,600 . | IgG and albumin | 2·25 | 25,800 | 41,600 . | IgG and albumin | 2·50 | 14,500 | 23,600 | |
| 4 . | No serum protein detected | — | — . | No serum protein detected | 3·62 | — | — . | No serum protein detected | 4·12 | — | — | |

IgA only. It was for this reason that it appeared essential to include the results obtained from both the calculations from Equation 2 and also from the calibration chart in Fig. 1. The molecular weight of the proteins in this peak varied between 400,000 and 626,000 even for normal human serum which is known to contain normal IgM of molecular weight of 900,000. The IgA in peak 1 may be a polymer or aggregate of IgA.

*Peak* 2.—As shown in Table III the proteins in this peak had M.W. between 140,000 and 230,000. The S.G. 200 peak 2 of normal as well as Burkitt urines contain both IgA as well as IgG. In one Burkitt patient's urine IgM was also detected in peak 2 and appeared to have a molecular weight in the range of 150,000 and 200,000. The precipitin lines of some Burkitt urine protein fractions formed against anti Fab and anti Fc showed reactions of complete identity (Fig. 2) suggesting that heavy polypeptide chain sub-units as well as intact IgG were present in this peak.

FIG. 3.—S.G. 200 filtration pattern of a normal human urine. The distribution of the immunoglobulins is indicated.

*Peak* 3.—The molecular weight of the proteins in this peak varied between 15,000 and 40,000. As shown in Table III the proteins present in the Burkitt urines seems to have the smaller molecular weight. IgG and albumin were the main proteins. In Fig. 2a and b the precipitin lines formed against anti Fab and Fc showed a reaction of non-identity probably indicating that light polypeptide chains as well as heavy polypeptide chains of IgG were present in this fraction.

*Peak* 4.—Although this peak did not appear to contain any protein, further tests seem necessary to ascertain this. Fig. 3 shows a typical gel filtration pattern of a normal urine.

*Immunodiffusion*

The immunodiffusion pattern of one of the Burkitt urines which gave a positive IgM precipitin line after gel filtration is shown in Fig. 2. Four patients with Burkitt's lymphoma contained IgM in their urines. In the first patient IgM was detected in both the concentrated whole urine as well as in the Sephadex G 200 peak 1. The urine of the second patient had IgM in peaks 1 and 2. In the third patient, IgM was detected only in the concentrated whole urine. The fourth urine was not fractionated but IgM was detected in the concentrated whole urine.

### DISCUSSION

The significance of the IgM in the urines of 4 out of 17 Burkitt lymphoma patients is not absolutely clear and needs further study. It is probable, however, that the excretion of IgM into both the urine and C.S.F. may contribute to the low levels of IgM which one so frequently encounters in the sera of Burkitt's lymphoma patients. There is a strong indication that this urinary IgM represents sub-units of the intact molecule. The degree of proteinuria in Burkitt's lymphoma patients is higher than in controls and may be the result of renal involvement. The characteristics of the urinary IgG and IgA in Burkitt's lymphoma appear to be similar to that found in the normal urine. Intact, as well as sub-units of IgG were found in both. The control, as well as the Burkitt's lymphoma, urines had some IgA molecules which appeared to be of larger molecular weight than the normal serum IgA. It is probable that this IgA is of renal origin and may be similar to exocrine IgA which also emerges from Sephadex peak in the void volume and has a molecular weight of the same order as that described by Hong *et al.* (1966) and Newcomb *et al.* (1968). On the other hand Portmans *et al.* (1967) suggested that fragments of immunoglobulins may aggregate under conditions employed in concentrating normal urine.

Six of 16 Burkitt's patients had markedly reduced serum IgG levels, and a total of 12 patients had reduced serum IgM level. Four of the patients with low IgG also had very low IgM. A recent article in *Lancet* (1969) stated that of 5 Burkitt lymphoma patients with reduced levels of serum IgG only one survived while all 5 with normal IgG levels survived. It is clear that further work is necessary to elucidate the dysgammaglobulinaemia that occurs in Burkitt's lymphoma.

---

### EXPLANATION OF PLATE

Fig. 2.—Photograph of a gel diffusion pattern (a), and its corresponding relevant line diagram (b) of an immunodiffusion pattern of S.G. 200 gel filtration peaks 1, 2 and 3 of Burkitt's urinary proteins.

*Peak* 1 shows precipitin lines against rabbit anti IgM and anti whole human sera.

*Peak* 2 shows lines against rabbit anti sera to Fab, Fc, whole human serum (wH) IgM, and IgA. He is the control human serum.

*Peak* 3 shows precipitin lines against anti-Fab, Fc and anti whole human serum.

Abbreviations
M = Anti IgM
A = Anti IgA
wH = Anti whole human serum
Hs = Normal human serum
Fab = Anti papain fragment of IgG with both heavy and light chains
Fc = Anti heavy chain fragment of IgG

2a

2b

McFarlane, Barrow, Ngu and Osunkoya.

This work was supported by a grant from the British Empire Cancer Campaign for Research which the authors gratefully acknowledge.

### REFERENCES

EVERALL, P. H. AND WRIGHT, G. H.—(1958) *J. med. Lab. Technol.*, **15**, 209.
FLODING, P. AND KILLANDER, J.—(1962) *Biochim. biophys. Acta*, **63**, 402.
HONG, R., POLLARA, B. AND GOOD, R. A.—(1966) *Proc. natn. Acad. Sci., U.S.A.*, **56**, 602.
*Lancet*—(1969) Leading Article, ii, 200.
LEACH, A. A., O'SHEA, P. C.—(1965) *J. Chromat.*, **17**, 245.
LOWRY, I. H., ROSEBOROUGH, H. J., FARR, A. L. AND RANDALL, R. J.—(1951) *J. biol. Chem.*, **193**, 265.
MANCINI, G., VAERMAN, J. P., CARBONARA, A. O. AND HEREMANS, J. F.—(1963) *Colloq. Protides biol. Fluids*, **11**, 370.
NEWCOMB, R. W., NORMANSELL, D. AND STANWORTH, D. R.—(1968) *J. Immun.*, **101**, 905.
NGU, V. A., MCFARLANE, H., OSUNKOYA, B. O. AND UDEOZO, I. O. K.—(1966) *Lancet*, ii, 414.
OSUNKOYA, B. O., MCFARLANE, H., LUZZATTO, L., UDEOZO, I. O. K., MOTTRAM FRANCES, C., WILLIAMS, A. I. O. AND NGU, V. A.—(1968) *Immunology*, **14**, 851.
OUCHTERLONY, O.—(1958) *Prog. Allergy*, **5**, 1.
PORTMANS, J. R., BLOCH, K. J. AND JEANLOZ, R. W.—(1967) *Clinica chim. Acta*, **17**, 229.
SCHEIDEGGER, J. J.—(1955) *Int. Archs Allergy appl. Immun.*, **7**, 103.
UDEOZO, I. O. K., BEZER, A. E., OSUNKOYA, B. O., NGU, V. A., LUZZATTO, L. AND MCFARLANE, H.—(1968) *J. Lab. clin. Med.*, **71**, 912.
WHITAKER, J. R.—(1963) *Analyt. Chem.*, **35**, 1951.